# Predictive Control-Based Completeness Analysis and Global Calibration of Robot Vision Features

**DOI:** 10.1155/2021/7241659

**Published:** 2021-12-09

**Authors:** Jingjing Lou

**Affiliations:** School of Mechanical and Electrical Information, Yiwu Industrial and Commercial College, Yiwu, Zhejiang 322000, China

## Abstract

This paper provides an in-depth study and analysis of robot vision features for predictive control and a global calibration of their feature completeness. The acquisition and use of the complete macrofeature set are studied in the context of a robot task by defining the complete macrofeature set at the level of the overall purpose and constraints of the robot vision servo task. The visual feature set that can fully characterize the macropurpose and constraints of a vision servo task is defined as the complete macrofeature set. Due to the complexity of the task, a part of the features of the complete macrofeature set is obtained directly from the image, and another part of the features is obtained from the image by inference. The task is guaranteed to be completely based on a robust calibration-free visual serving strategy based on interference observer that is proposed to complete the visual serving task with high performance. To address the problems of singular values, local minima, and insufficient robustness in the traditional scale-free vision servo algorithm, a new scale-free vision servo method is proposed to construct a dual closed-loop vision servo structure based on interference observer, which ensures the closed-loop stability of the system through the Q-filter-based interference observer, while estimating and eliminating the interference consisting of hand-eye mapping model uncertainty and controlled robot input interference. The equivalent interference consisting of hand-eye mapping model uncertainty, controlled robot input interference, and detection noise is estimated and eliminated to obtain an inner-loop structure that presents a nominal model externally, and then an outer-loop controller is designed according to the nominal model to achieve the best performance of the system dynamic performance and robustness to optimally perform the vision servo task.

## 1. Introduction

The ability of humans to observe things in the outside world with both eyes and manipulate what we see is the result of a long evolutionary process that is important for us to adapt to and make use of the outside world. Inspired by this phenomenon, visual sensing was introduced to robot control systems to sense the state of the system and the task. This type of information is collected from the outside of the control system, compensating for the information fed back from within the system, relaxing the requirements for mechanical accuracy and joint tightness of the robot components, and allowing the robot system to enhance its flexibility in an uncertain external environment for various automatic control functions, such as robotic assembly, robotic surgery, and remote-control tasks. As a result, visual servo control, that is, the introduction of visual information into the control closed loop to guide dynamic system motion, has received a great deal of attention. The development of robotics is changing rapidly; robots are more functional and more versatile; not only traditional robots for industrial applications but also more military and medical robots along with the wave of artificial intelligence robots are making a splash in various fields [1]. Whether the information contained in the macro- and microfeatures is complete affects the completion of the task. Therefore, the complete visual feature set of the visual servo task is defined to describe the feature set that meets the needs of completing the task. The feature set consists of a complete macrofeature set and a complete microfeature set. The wide application of robots makes robotics products form a huge industry. At the same time, the development of robotics will have a huge and far-reaching impact on the country's comprehensive national power and its ability to develop sustainably. Therefore, robotics is not only seen as a frontier technology but also taken as a national strategy by many developed countries in the world, which gives strong support to the research in this field. As the most important class of robots, mobile robots will be used in industry, agriculture, national defines, scientific research, and other industries to partially or completely replace the work of people as human society continues to develop and the degree of information technology and intelligence continues to increase.

Vision-based mobile navigation is a research hotspot, and research in this area can currently be divided into three parts: visual simultaneous localization and mapping (VSLAM), path planning, and navigation control. Over the past decades, a large amount of research has focused on VSLAM, which is the study of environmental modelling. The process requires the system to build the environment while using the known local environment to achieve self-localization and then incrementally model the environment [2]. Research in this area, often without considering the command generation for its movement control, but only how to achieve environment reconstruction as well as real-time localization is given the information, is essentially a real-time version of structure from motion (SfM) in the field of computer vision [3]. In the visual navigation process, the captured image sequences are used to achieve online incremental environment perception and autonomous localization using VSLAM. The concept of feature completeness for robot vision servo tasks is proposed, and the complete feature set is defined to consist of a complete microscopic feature set and a complete macroscopic feature set. For an arbitrary vision servo task, a criterion is given to judge whether a microscopic feature set is complete or not, and three metrics are proposed to evaluate the performance of a complete microscopic feature set. The task of grasping a brush by a humanoid robot is taken as an example to study the acquisition and application of the complete microfeature set in the robot vision servo task [4]. According to the proposed definition and discriminative quasi-discrimination of the complete feature set, the complete feature set is designed. The camshaft algorithm is used to detect target points and actuator ends in the video stream captured by the system camera, and then the complete feature set is obtained.

Mobile robots often work in unstructured and unknown environments and need to autonomously sense and perceive the environment according to specified task goals and plan their behaviour efficiently and intelligently to accomplish specific tasks. To accomplish tasks more efficiently, mobile robot systems contain a central decision-making system and several subsystems that are responsible for different subtasks [5]. Among them, autonomous navigation technology for mobile robots is a fundamental module. The navigation system is the minimum complete system integrating environment modelling, scene understanding and reasoning, autonomous decision-making and execution, and human-robot interaction, corresponding to a complete processing process of information representation, association, fusion, reasoning, and management. Therefore, the level of intelligence of a robot navigation system also determines the complexity of the environment it can adapt to and the complexity of the navigation task. The intelligence level of robot autonomous navigation systems is closely related to the corresponding methods of information processing, feature expression, and autonomous cognition. In recent years, the leap in technologies such as high-speed cameras, image processing hardware, and high-performance mobile computing platforms has provided mobile robots with the assurance of perceptual information and computing efficiency for autonomous navigation. The rapid development of information intelligence processing technology also keeps pushing the robot autonomous navigation system towards the direction of intelligence, which makes the robot gradually improve its adaptability and autonomy to the environment. This paper focuses on the effect of different feature sets on the completability and completion performance of an uncalibrated visual servo task. Feature completeness is proposed to examine whether a feature set provides enough information to make a task complete. Evaluation metrics are proposed to examine whether a completeness feature set enables optimal task completion. The humanoid robot grasping brush task and the robot writing Chinese calligraphy task are used as examples to investigate how to select, extract, and use the complete feature set for different visual servo tasks, respectively. Based on this, a robust calibration-free visual servo control method based on the complete feature set is proposed to improve the task completion performance.

## 2. Status of Research

Since the control commands of the vision servo system are calculated based on the hand-eye mapping relationship, the transient performance, steady-state performance, and stability of the system are affected by the hand-eye relationship [6]. The calibration-free vision servo system uses visual information to guide the robot's motion when the camera parameters are not calibrated. How to obtain an accurate hand-eye relationship in uncalibrated vision servo control is an important research problem [7]. The hand-eye mapping relationship for visual servo systems is strongly nonlinear and can be approximated by a series of linear relationships within each local domain, such as the use of image Jacobian matrices. There are two main ways to calculate the image Jacobian matrix, indirectly or directly, to estimate the matrix take values. One way to estimate the image Jacobian matrix indirectly is by estimating the unknown parameters in the model. The literature [8] represents the uncalibrated visual servo problem as a multiple-input, multiple-output adaptive control problem and accomplishes visual servo control using a Lyapunov adaptive control method based on SDU decomposition with online calibration of the camera parameters through a cascade control strategy. The literature [9] investigates how a system with unknown camera and robot parameters can accomplish calibrated control. The algorithm uses a depth-independent image Jacobian matrix to establish a mapping of visual deviations to the joint angle space of the robot arm so that the unknown camera and robot parameters appear linearly in the closed-loop dynamic structure of the system, which in turn estimates the unknown parameters online using an adaptive algorithm [10]. Another approach estimates the image Jacobian matrix directly using a different method.

The spatial projection of image pixel points using multiview geometry as well as feature point matching and optical flow tracking is used to obtain a spatial point cloud, and the matching of this point cloud map with the current image and the PnP algorithm is used to localize the robot system [11]. Its localization optimization is performed with the reprojection error or image error as the optimization term for the pose solution. The method is characterized by high operable accuracy and intuitive map representation [12]. The basic dual-view geometric relative pose solution usually uses feature points to represent the image and calculates the basis matrix or single response matrix between two frames by matching the feature points and then obtains the relative pose between two frames by decomposing the singular values of the matrix [13]. The method has a deep theoretical foundation and can obtain pose calculation results with high accuracy under the premise of having ideal data matching [14]. This kind of reprojection method is usually implemented based on feature points, so this kind of method relies on the information of individual pixels in the image for localization, and this kind of matching method with local information often limits the reliability of data matching due to the limitation of its observation window size; in addition, the amount of information used in the selection and expression of pixels is limited, so this method has a weak ability to overcome changes in environmental conditions. In addition, due to the limited amount of information used, the ability to overcome changes in environmental conditions is weak, resulting in problems such as data matching errors or insufficient effective matching [15]. These problems also lead to the inability of visual navigation based on these methods to achieve reliable localization output and stable navigation under a long period or harsh working environment, which reduces the robustness of the visual navigation system.

Real-time modelling for the unstructured environment in the human-robot collaboration scenario can provide global process information for subsequent motion planning, which is a key technology for visual perception. It mainly studies the influence of different feature sets on the accomplish ability and completion performance of an uncalibrated visual serving task. The feature completeness is proposed to investigate whether a feature set provides enough information to make a task complete. According to the above comparison, the depth camera based on the principle can calculate the depth information in real-time and fast, with moderate accuracy and a large field of view, which can fully meet the robot's needs for motion planning in a large working space. However, after analysis, in unstructured working scenes, the field of view of a single depth camera is affected by obstacle occlusion, so it is necessary to introduce multiple depth cameras to form a high real-time global vision system to obtain more complete environmental information and improve the success rate and safety of motion planning. Therefore, efficient modelling of unstructured environments is one of the main research directions of this paper.

## 3. Predictive Control for Robot Vision Feature Completeness Analysis and Global Calibration Analysis

### 3.1. Predictive Control for Robot Vision Feature Completeness Design

Visual servo tasks require optimization of the overall performance of the task from a macroscopic perspective and the achievement of planned control goals at each step from a microscopic perspective. Both macroscopic optimization and microscopic control require description and feedback of the task in terms of visual features [16]. The completeness of the information contained in the macro- and microfeatures affects the completability of the task. Therefore, the complete set of visual features for a visual servo task is defined to describe the set of features needed to satisfy the task completion, which consists of a complete macrofeature set and a complete microfeature set. Macrofeatures are often built on top of microfeatures, so this section discusses the complete microfeature set first. Robot vision servo control is an interdisciplinary research field, involving multiple disciplinary elements such as image processing technology, computer vision, control theory, and real-time computing. However, the purpose of its task execution is to achieve control goals in the task space. Microscopic visual features play a role in visual servo control to characterize and feedback the control condition of the system. Therefore, evaluating the merit of a microscopic visual feature set for a visual servo task examines whether the hand-eye relationship adequately links up control and visual features. That is, the microscopic visual features should be sufficiently informative to ensure that every control degree of freedom is characterized and fed back by the visual features.

(1) $$\begin{array}{c} f=\frac{\partial H\left(q\right)}{\partial q}\dot{q},\\ f=J\cdot U^{2}. \end{array}$$

The image Jacobian matrix varies with robot poses, so for a particular set of microscopic features, it is important to ensure that the image Jacobian matrix is a full rank within the robot motion range involved in the task. Based on ensuring that the visual servo task can be completed, the feature set with the best properties is selected so that the task completion effect is optimized. First, the mapping relationship between the image space and the task space should be as stable as possible, changing substantially as the relative poses between the target object and the camera change so that the feature set can avoid the system converging to local minima or having unintended motions in the task space [17]. Second, the rate and magnitude of change of the microscopic visual features should be as consistent as possible with the control volume to better characterize it. The microscopic features are chosen to avoid singular values and local minima as much as possible. When singular values or local minima are present, both visual feature changes and control volume changes will be extremely inconsistent. At singularities, it is known from the hand-eye mapping relationship that a nonzero velocity vector at the end of the actuator corresponds to a visual feature deviation that may be zero, and thus, the visual feature does not correctly reflect the condition of the controlled robot. At local minima, the feature deviations belong to the zero space of the generalized inverse matrix of the image Jacobian matrix.

(2) $$\begin{array}{c} f+f^{*}=\mathrm{Ker}\;J^{+}. \end{array}$$

**Figure 1:**
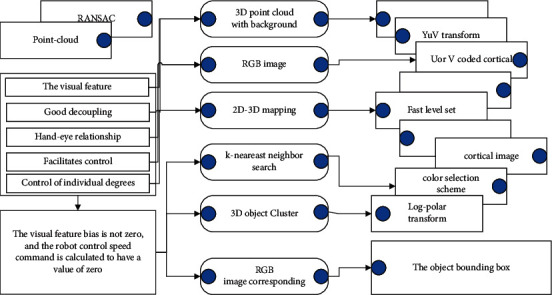
Robot vision feature completeness framework.

The visual feature bias is not zero, and the robot control speed command is calculated to have a value of zero, so the visual feature cannot be used to guide the control either. In addition, good decoupling of the hand-eye relationship facilitates control and planning to avoid control of individual degrees of freedom causing undesired operations in other degrees of freedom, resulting in reduced control efficiency or actuator end motion out of the field of view, as shown in Figure 1.

For the human-machine collaborative operation system, there is a state that the human-machine is not in the same space at the same time; then the robot's working space is an unstructured environment occupied by static obstacles. Unlike the free space or structured environment where the state is known, the work objects and obstacles in the unstructured environment are irregular and randomly placed, especially for the customized and small-batch operations where the human-robot collaborative production method is applicable; the work objects are of many types and often change production. The navigation system is the smallest complete system that integrates environment modelling, scene understanding and reasoning, autonomous decision-making and execution, and human-computer interaction. It corresponds to a complete processing flow of information expression, association, fusion, reasoning, and management. In the actual operation process, the robot first needs to identify the target workpiece among multiple types of work objects, that is, to achieve the position estimation of the target workpiece, which is the basis for completing the subsequent operation tasks and is exactly what was studied in the previous section of this paper [18]. In the process of executing subsequent tasks, the robot calculates the corresponding end tool posture based on the workpiece posture and moves safely and autonomously to the target posture in an unstructured environment, which is an important manifestation of the robot's intelligence capability, and motion planning is one of the key technologies to realize the robot's autonomous motion, which is also the focus of this chapter. Since robots for human-robot collaboration usually have six or seven degrees of freedom, their planning space is a complex high-dimensional space, which leads to complex space modelling and increased computational effort for collision detection, affecting the efficiency and success rate of motion planning. To solve the above problems and quickly plan feasible motion paths, this chapter first introduces the planning principle of the classical fast extended random tree algorithm and then proposes a heuristic-guided fast extended random tree algorithm for its problems of randomly scattered sampling points and slow convergence, which uses a guided search strategy to accelerate the convergence of the algorithm, and heuristic functions are used to optimize the motion paths to make the optimal motion path length.

(3) $$\begin{array}{c} J=\left[\begin{array}{ccc} \frac{\lambda_{1}}{z_{1}} & 1 & -\frac{f_{1}}{z_{1}}\\ 1 & \frac{\lambda_{1}}{z_{1}} & -\frac{f_{2}}{z_{1}}\\ \frac{\lambda_{1}}{z_{1}} & 1 & -\frac{f_{3}}{z_{1}} \end{array}\right]. \end{array}$$

The camera of a specific focal length can be selected according to the range of features in the actual experiment to avoid singular Jacobian matrices when the inverse matrix zero space of the image Jacobian matrix contains only zero vectors and avoids local minima. Thus, the feature set consisting of the pixel coordinates of the marker points is the complete feature set for this task. With the feature set complete, its performance is discussed. The image Jacobian matrix does not change rapidly with the relative positional change of the hand-eye, so the linear nature is acceptable. Good consistency of hand-eye variation is obtained by avoiding singular values and local minima with a suitable choice of camera focal length. There are three zero terms in the image Jacobian matrix, so the hand-eye relationship under this feature set is well decoupled. The simplicity of the points makes the feature set acquisition efficient and satisfies the requirement of processing speed in human-computer interaction.

(4) $$\begin{array}{c} \theta_{0}=\arctan\left(\frac{2\left(\left(M_{11}/M_{00}\right)+x_{\lambda }y_{\lambda }\right)}{\left(\left(M_{11}/M_{00}\right)+x_{\lambda }^{2}y_{\lambda }^{2}\right)+\left(\left(M_{11}/M_{00}\right)-x_{\lambda }y_{\lambda }\right)}\right),\\ f\left(k-1\right)=f\left(k\right)+J\left(q\left(k\right)\right)\cdot \Delta q\left(k_{k}^{2}\right). \end{array}$$

**Figure 2:**
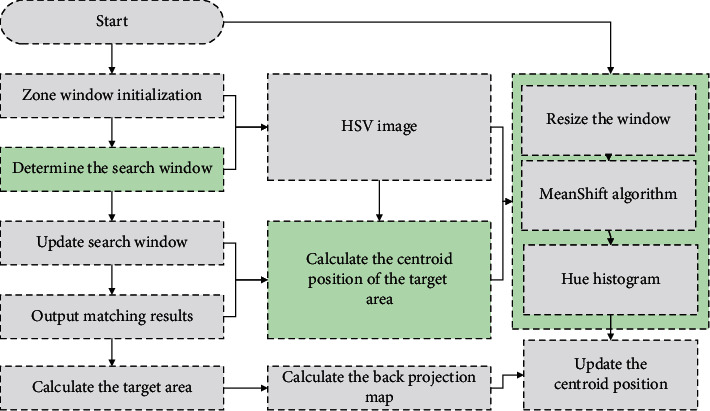
Predictive control steps.

The autonomous navigation behaviour of a mobile robot is first based on stereo vision sensors to obtain information about the surrounding environment and construct an environmental possibility map and plan a reasonable executable path under the a priori information of the possibility map, and finally, the robot follows this executable path to complete the obstacle avoidance navigation task [19]. The key to the robot navigation problem is to be able to find a safe and smooth path in the process of moving towards the target point while enabling the mobile robot to accomplish the task of dynamic obstacle avoidance. The existing mobile robots have a certain autonomous navigation capability, but because the characteristics of the field environment are more complex and dynamic, the control error and measurement error of the mobile robot is greater; therefore, it is necessary to conduct further in-depth research on the key technology motion planning of the autonomous navigation behaviour of the mobile robot in the field environment, as shown in Figure 2.

For the visual tracking constraint, since this kind of constraint is performed between the current frame and the previous keyframe with a short visual tracking baseline, it can be assumed that the relative motion between the two images is small, and it is feasible to use the optical flow method for data matching alignment in that case. Three indicators are proposed to evaluate the performance of a complete microfeature set. Taking the task of a humanoid robot grabbing a brush as an example, the acquisition and application of a complete set of microfeatures in the task of robot visual serving are studied. Also, it is appropriate to use the optical flow-based method to handle the visual tracking constraint calculation with a higher establishment frequency because of the simple and efficient data matching process. Based on the matching results of the optical flow method in the keyframe selection stage, pixel matching between two frames can be simply obtained to complete the data association establishment of visual tracking constraints.

To avoid the computationally expensive problem of too many candidate regions due to probabilistic sampling, the sampling mode of other traditional tracking algorithms is replaced by a local search cantered on the location of the target, which reduces the search range and improves the computational efficiency. The advantage of local search is that it considers the continuity of the target's motion trajectory and the smoothness of the appearance transformation during the tracking process. The previous frame filter model, obtained by using the filter template update method, is correlated with the local search region of the current image frame to locate the position of the tracking target in the current frame.

(5) $$\begin{array}{c} y=F^{-1}\left\{Z\;⊕\;F^{*}\right\}. \end{array}$$

To introduce multiscale estimation in the algorithm to obtain an accurate extraction of the region where the target is located, but not to lose the rapidity of tracking efficiency due to the increase in computational complexity, an incremental estimation strategy is used. The strategy separates the position estimation from the scale estimation, forming a cascading relationship. After relying on position estimation to capture the location of the target from the image, scale estimation is enabled to extract the extent of the target's region in the image. This estimation strategy does not affect the computational process of location estimation but also relies on its results to reduce the scale estimation range and reduce the computational cost.

### 3.2. Experimental Analysis of Visual Feature Completeness Bureau Calibration

Based on the positional information of the operation object, the robot needs to plan a feasible path in the unstructured environment. Since in unstructured scenarios such as human-robot collaboration, there is a situation where the human and the robot are in the same workspace at the same time when the robot is in a dynamic unstructured scene, the state changes of the human, the robot, and the environment are random, so the robot needs to intelligently sense the state changes of the obstacles in the workspace through vision sensors, and when the obstacles may interfere with its normal operation, the robot needs to react in time, that is, plan its motion trajectory online to achieve obstacle avoidance to avoid potential collision hazards [20]. Therefore, this chapter proposes an online real-time trajectory planning method for robots in dynamic unstructured environments.

**Figure 3:**
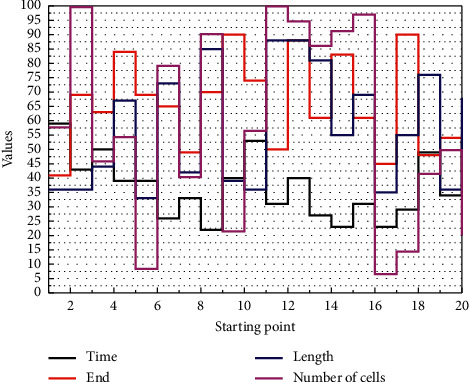
Path planning.

First, to solve the problems of the limited single-camera field of view and real-time dynamic obstacle avoidance, a visual perception method is proposed to establish an offline mapping and online fusion model of multicamera depth images and robot workspace 3D raster, determine the occupation state of the occluded 3D raster by the robot and obstacles, and obtain the nearest distance and relative velocity between the stable robot and obstacles in real-time based on Kalman filtering algorithm. It often does not consider the generation of commands for movement control but only considers how to achieve environmental reconstruction and real-time positioning under given information, which is essentially a real-time version of the field of computer vision. Then, based on the improved artificial potential field method to calculate the attractive and repulsive forces, the trajectory avoidance strategy is adjusted according to the relative position and velocity of the obstacles, and the potential field forces are converted into robot joint velocities to control the robot from the velocity level to complete the obstacle avoidance task so that the robot can avoid obstacles and ensure the operational efficiency at the same time. Finally, the effectiveness of the trajectory avoidance algorithm is verified in scenarios with different dynamic obstacles, as shown in Figure 3.

Unlike current path planning methods that optimize the path search approach to improve efficiency, this chapter improves the efficiency of path search by reconstructing the environment representation model. The multilevel graph model-based environment representation method can describe the multilevel topological structure information of the environment more effectively, which in turn improves the efficiency of using environment-inspired information in the path search process. This experiment further validates the idea that building a task-oriented environment representation model can improve the efficiency of retrieving and utilizing information during task execution. However, in the path tracking process, to track the feature paths, the mobile robot needs to accurately identify the global features of the environment, thus requiring higher performance of global localization. The mobile robot needs to globally localize not only its pose but also the features of the environment.

**Figure 4:**
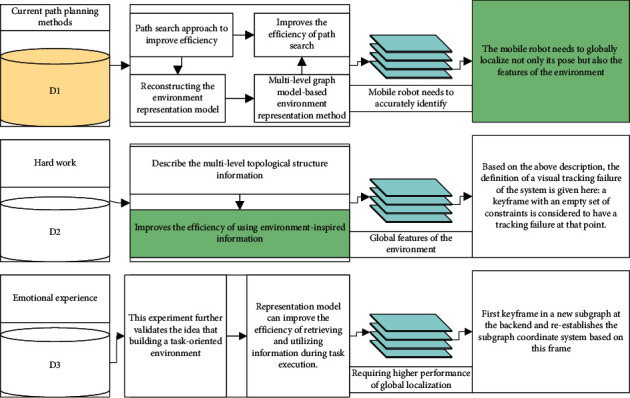
Back-end flow diagram based on multiple subgraphs.

Based on the above description, the definition of a visual tracking failure of the system is given here: a keyframe with an empty set of constraints is considered to have a tracking failure at that point. When a tracking failure occurs, the system considers this keyframe as the first keyframe in a new subgraph at the back end and reestablishes the subgraph coordinate system based on this frame so that future visual tracking is maintained in this new subgraph. It is important to note that in contrast to the first keyframe initialized by the system that is cured and not involved in the optimization adjustment, the first node of the newly created subgraph here is not cured and will be involved in the future global graph optimization adjustment process. With this subgraph mechanism, the system will always be in two states: maintaining visual tracking in a subgraph or creating a new subgraph and trying to track maintenance in that subgraph. Thus, the system can run all the time without running out. The flow diagram of the back end of this subgraph is shown in Figure 4.

During the visual tracking of a particular subgraph, the poses of the nodes are given according to the motion model. The pose estimate of the current image frame is determined by the known pose of the related frames within the same subgraph and the constraints between the two frames. Also, nodes that are within the same subgraph will be influenced by other nodes that are constrained by another subgraph, thus achieving an overall alignment of the subgraph. Mobile robots are the most important category of robots. With the continuous development of human society and the continuous improvement of informatization and intelligence, mobile robots will surely replace human work partially or completely in industries such as manufacturing industry, agriculture, national defense, and scientific research. Thus, visual tracking always operates within a certain subgraph, and the operation of that visual tracking is always a two-view geometric operation based on two frames of images. With such a strategy, it is possible to maintain the operation of the system in the face of visual tracking failure in this system, despite the loss of information about other existing nodes.

When building a workstation for human-robot collaboration, the area of interest to be observed needs to be set and multiple cameras need to be arranged according to the scope of the working scene regarding the above method to obtain more complete environmental information [21]. Although two cameras can acquire more complete environmental information, the amount of data acquired by two depth cameras at the same time is large, and they need to be updated and fused in real time, which requires more computational effort and time, resulting in the robot's intelligent perception and trajectory planning performance in dynamic unstructured environments being affected by the online updating efficiency of the environmental model. Therefore, to speed up the fusion and update of dual-camera data, this paper draws on the idea of offline modelling and online updating, builds a model of the projection relationship between 3D raster and depth image in the offline stage based on the projection principle of 3D space and 2D image, calculates the projected pixel points of each 3D raster centre in the robot workspace on the depth image, and constructs the mapping data structure of 3D raster to depth map pixels to the data set that is saved offline.

## 4. Results and Analysis

### 4.1. Predictive Control Results for Completeness of Robot Vision Features

**Figure 5:**
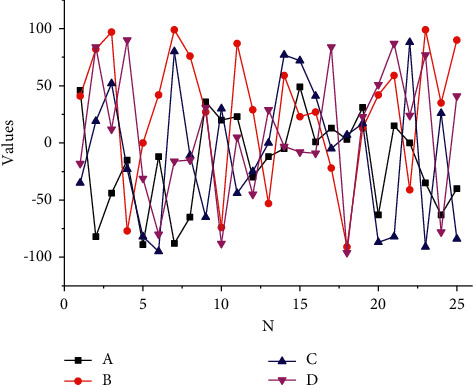
Observed tracking bias.

Figure 5 depicts the entire process of right-hand tracking for the NAO robot. In this task, the binocular vision system takes pictures of the workspace and the vision servo system uses the Camshafts algorithm to detect the end of the actuator and the quill. In this way, this process of the task can be described in vision space. At the beginning of the task, the NAO robot's left and right cameras capture images with a difference of 111 pixels in the horizontal and 163 pixels in the vertical direction. A Kalman–Bucy filter-based visual servo controller is used to estimate the image Jacobian matrix, which in turn derives control quantities to act on the robot. The NAO robot eventually reaches the quill within 17 steps, and the deviation between the two centres is within 10 pixels. The experimental results show the robustness of the algorithm proposed in this paper to image noise, motion disturbances, and irregular motion of the target object.

Task-oriented evaluation of visual feature sets examines the impact of feature sets on task completability and completion performance. First, a definition of a complete feature set for a visual servo task is proposed, and a complete feature set includes a complete microfeature set and a complete macrofeature set. This section focuses on the complete microfeature set and proposes a criterion to determine whether a feature set is a complete microfeature set for a visual servo task. On this basis, if the visual feature set is complete, its performance needs to be further examined in terms of the linear nature, change consistency, and decoupling of the complete feature set. Taking a humanoid robot grasping a brush as an example, the acquisition and use of the complete microscopic feature set is investigated, and a visual servo system is proposed, which is built based on the calibration-free Kalman–Bucy filter visual servo method and the Camshaft detection algorithm. Using this system, the NAO robot accomplished the grasping of brushes well in the calligraphy task. The experimental results verify the validity of the theory of complete microscopic feature sets proposed in this chapter and the robustness of the complete feature set extraction algorithm to image noise, humanoid robot return error, and an irregular motion of the target object.

**Figure 6:**
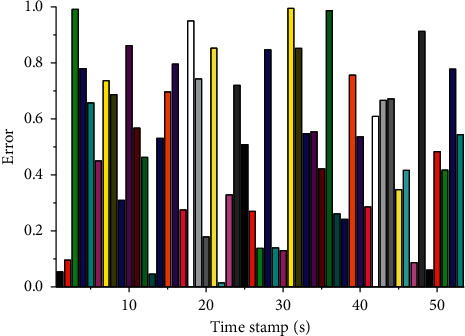
Analysis of positioning error accuracy at the back end of multisubgraph.

In terms of quantitative evaluation for the multisugar back end, to demonstrate the improved enhancement of this back end design for fault recovery, we recorded the timestamps of the occurrence of tracking loss and closed-loop detection during the experiment, as well as the localization error at the corresponding time points. The results are shown in, Figure 6, where the difference between the true bit poses recorded by the red grasping system is obtained, the light blue line is the response marker for the moment of tracking loss, and the red line is the response marker for the occurrence of closed loop. During the figure, visual tracking, as well as map construction, can always maintain operation in either subgraph, and when mutual constraints are detected between the subgraphs, the error curve decreases due to the alignment between the subgraphs. Furthermore, the trend of the error curve shows that after a certain time of environmental exploration, the error curve stops rising, and any new keyframe always finds a relevant reference frame in the map, which in turn establishes a constraint and achieves successful localization. Such curve results quantify the ability of the system in tracking lost fault recovery.

The decreasing trend in the number of particles employed in the process indicates that the robot positional uncertainty is reducing the influence of its fluctuation process by the environmental structure. In the algorithm of this chapter, the number of effective particles is always maintained at a low level, which makes the algorithm require a low amount of computation. As the mobile robot moves, it collects enough information about its environment, and the uncertainty in its positional estimation decreases. However, to still ensure the robustness of the positional estimation, the algorithm still maintains a certain number of particles for the estimation of the positional pose.

The convergence rate is low in the corridor scenario because only the boundary information of the motion model is provided and the inner content is not fully described. Also, due to the use of omnidirectional observation particles, while obtaining as many possibility regions as possible, it introduces correlation uncertainty that needs to be fused with more observations for elimination. From the comparison experiments, the initial likelihood region calculation has an important impact on the convergence speed of the global localization algorithm. In the corridor scenario, it is difficult to make an accurate estimate of the region in which the initial poses are located. However, in the results of the method in this chapter, despite the large deviation of the initial moment's positional estimation, the algorithm is still able to converge to the correct region after a period of motion, which fully verifies the robustness and stability of the method in this chapter in the process of global localization. It compensates for the information fed back from the system, relaxes the requirements for the mechanical accuracy and joint tightness of the robot components so that the robot system can also improve flexibility in an uncertain external environment, and realizes various automatic control functions. Through simulation experiments, this chapter verifies the effectiveness of the proposed method in terms of global localization accuracy and further verifies the effectiveness of this chapter's method in terms of global localization efficiency and global localization robustness through online experiments.

### 4.2. Experimental Results of Visual Feature Completeness Bureau Calibration

Geometric features in scenes are pervasive features, less affected by texture, more robust to changes in perspective, and so on. Geometric features can improve the ability to represent space. Establishing a mapping model of geometric feature descriptors to their spatial locations and constructing a method to retrieve their corresponding geometric features for a given spatial location are essential to improve the ability of geometric features to represent space. There are two main types of geometric features. One is the features corresponding to geometric primitives, such as line segments, planes, and other features. The second is local geometric features of dense surfaces, which do not have a specific form of expression but describe geometric information of the corresponding surface and can generally be modelled using surface elements. Single features have limited ability to characterize spatial information and are more dependent on environmental conditions. Exploring global localization methods for multiple features is important for improving navigation system stability and environmental adaptability.

**Figure 7:**
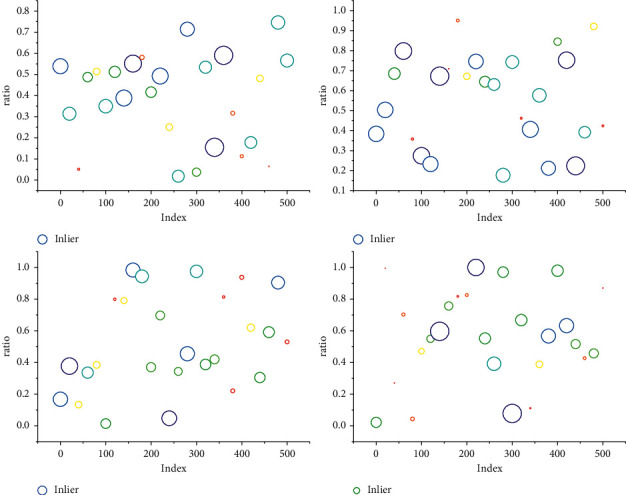
Results of point matching within the test image.

Line segment features are pervasive features in the environment and are the main environmental features in weakly textured scenes. Their number is generally small, but the feature descriptors are relatively expressive. In this chapter, a visual global localization method based on multivariate geometric features is proposed, which can better reason about feature associations by using the relationship between multivariate features. Furthermore, since the dense geometric information of the environment can greatly improve the screening quality of the positional hypothesis, this chapter explores more efficient dense map representation methods that enable the robot to quickly retrieve location-specific geometric distribution information to speed up the screening efficiency. A schematic diagram of visual relocation based on multivariate geometric features is shown in Figure 7.

**Figure 8:**
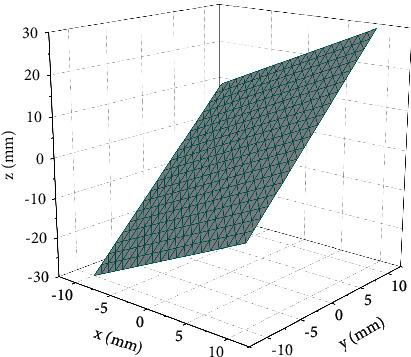
Tracking trajectory obtained using interference observer-based vision in a workspace.

In the interference observer-based visual servo control, the tracking results observed from both cameras are shown in Figure 8. It can be seen from the figure that the system can track the target object well using the interference observer-based algorithm even in the presence of image noise and external interference. It can be concluded that the visual servo method based on interference observer can suppress the effect of image noise and external interference well while ensuring the closed-loop stability of the system. At the same time, there is no steady-state error.

The experiments were first conducted using a pioneer robot equipped with vision sensors to collect training data and test data in a laboratory scene and a corridor scene. The lab scene contains richer texture information, while the corridor scene is a typical weak texture scene. Line segment features are the main feature type in the corridor. The data sequences collected in the static laboratory scene are Official and Office 2. To test the robustness of the method in this chapter to dynamic objects, pedestrians are set to walk during the test, and the corresponding data sequences are Office-D and Corridor-D. The method in this chapter is compared with the DBoW2 method, which is a global localization method widely used in current SLAM systems. It can be seen from Figure 8 that the method in this chapter can obtain more successful localization frames, in which, the indexes of the training and test sequences are given in the table. The experimental indexes are the number of frames with localization error less than 5 cm and the number of frames less than 10 cm. In the laboratory scenario, more correct localization frames can be obtained due to the richer features. Comparing the localization results of ORB-SLAM2 in Office and Office-D, the robustness of the method in this chapter is higher for dynamic objects. Although the method in this chapter obtained fewer successful localization frames with errors less than 5 cm in the corridor scene, most of the frames were localized with errors of 10 cm, indicating the robustness of the method in this chapter for dynamic, low-texture scenes.

The feature point maps, line segment feature maps, and dense point cloud maps learned through regression trees, which greatly improve the efficiency of feature-based retrieval of space and spatial information-based retrieval of features. In the process of global localization, the camera poses are estimated by combining the applied point information and line segment information, and stable point pair matching is constructed using point-to-linear projection to realize the computation of positional assumptions. The stacked RANSAC method is constructed to address the problem of insufficient sampling efficiency due to the low proportion of interior points in the matched pairs. Compared to the traditional RANSAC method, the stacked RANSAC method uses the results of the previous sampling information to guide the new sampling process to be more biased towards meaningful samples. At the same time, a more efficient method of assuming a quality metric for the poses is utilized to constrain the current observation to match the environmental surface in a more efficient manner using the dense geometric information of the environment.

## 5. Conclusion

The concept of a complete feature set is proposed from the perspective of visual servo task completability; the determination method of a complete microscopic feature set is given; and the extraction and use of a complete microscopic feature set are investigated using a humanoid robot grasping a brush as an example. In this paper, the hand-eye relationship of the feature-based 2D visual servo task is described by the image Jacobian matrix, and the completeness of the microscopic feature set is examined by finding the rank of the image Jacobian matrix to see whether the microscopic feature set completely characterizes the robot control degrees of freedom, that is, the completeness of the microscopic feature set is examined. The camshaft method is used to track the target object, obtain the microscopic feature set, and complete the task based on the complete microscopic feature set using a Kalman–Bucy filter-based visual serving method. Through the experiments based on the NAO robot, it is confirmed that the feature set proposed in this paper can guarantee the completion of the task, the microscopic feature set acquisition method has good robustness in the presence of image noise and irregular motion trajectory of the target object, and finally, the visual serving task can be completed with good performance according to the whole visual serving strategy. Based on the Q-filter to construct the interference observer, the equivalent interference consisting of model uncertainty, high-frequency detection noise, and low-frequency input interference is estimated and eliminated through the input and output signals of the controlled object so that the part consisting of the controlled object and the interference observer externally exhibits the given nominal model, and then the visual servo controller is designed according to the nominal model to achieve robustness under the premise of closed-loop stability of the system high-performance control effect under the premise of closed-loop stability of the system. Finally, the effectiveness of the calibration-free visual servo method proposed in this paper is verified by comparing the algorithm with the proportional-integral method and the divisional Brayden method in the simulation environment and comparing the method with the proportional-integral method in the experimental conditions.

## Data Availability

The data used to support the findings of this study are available upon request to the author.
